# Upregulated Expression and Shifted Distribution of Melatonin and Its Synthesizing Enzymes From Postnatal to Young Adult Rat Cochleae

**DOI:** 10.1002/dneu.22979

**Published:** 2025-06-18

**Authors:** Monika Orsolic, Marc Diensthuber, Timo Stöver, Christin Geißler

**Affiliations:** ^1^ Goethe University Frankfurt University Hospital Department of Otorhinolaryngology Frankfurt am Main Germany

**Keywords:** AANAT, cochlea, HIOMT, inner ear, melatonin

## Abstract

The cochlea's cellular architecture plays a critical role in auditory perception, yet is highly susceptible to degenerative factors. While melatonin is known for its antioxidative properties in the adult cochlea, its expression during early development remains understudied. This study used immunohistochemical staining of melatonin and its synthesizing enzymes (AANAT, HIOMT) to explore the self‐synthesis and spatial distribution of melatonin in the cochlea of postnatal and adult rats. Postnatal rats exhibited low levels of intracellular marker expression in the lateral wall and the undifferentiated sensory epithelia, with no expression observed in the spiral ganglion. They showed mainly extracellular marker expression near the stria vascularis, in the stria vascularis interspace, and above undifferentiated sensory epithelia. In contrast, adults exhibited widespread intracellular marker presence in spiral ligament fibrocytes, spiral ganglion neurons, satellite glia, and epithelial supporter cells, except in hair cells. Fibrocytes in the spiral limbus expressed AANAT and HIOMT at both developmental stages. The present findings indicate melatonin's complex involvement in cochlear protection and development. Detailed knowledge of melatonin synthesis locations and intensity across different age stages within the auditory system holds the key to pioneering novel treatments, preventive strategies, and a deeper understanding of hearing processes.

## 1 Introduction

The cochlea of the inner ear contains structures that transform acoustic waves into electrical signals essential for hearing. Within the cochlea, hair cells detect sound vibrations and generate electrical impulses. These impulses are relayed to the spiral ganglion neurons, which then transmit the signals to the brain via the auditory nerve. The cochlea is vulnerable to deterioration, from age to the harshness of environmental factors and drugs (Bas et al. 2009; Demir et al. 2015; Serra et al. 2020).

Given this vulnerability, the emerging understanding of melatonin offers promise in terms of protecting the cochlea. Originally identified as a pineal hormone, melatonin has been found to exert antioxidant, anti‐inflammatory, and anti‐glycolytic effects (Hardeland and Poeggeler 2012).

Melatonin has antioxidant, anti‐inflammatory, and anti‐glycolytic effects (Hardeland and Poeggeler 2012; Acuña‐Castroviejo et al. 2014; Sarlak et al. 2013) and modulates the extracellular matrix (He et al. 2014; Kaczmarek‐Szczepańska et al. 2021; Rudra et al. 2013; Ganguly and Swarnakar 2009). Furthermore, externally added melatonin is known to be protective for the inner cochlea and auditory function (Serra et al. 2020; Bas et al. 2009; Demir et al. 2015; Bas et al. 2012; Karaer et al. 2015). Nevertheless, melatonin remains insufficiently studied in the inner ear. The present study investigated the specific localization of self‐synthesis of melatonin in the inner ear at the cellular level based on the immunohistological detection of the enzymes required for its metabolism. The intermediate N‐acetyl‐serotonin (NAS) is synthesized from serotonin by serotonin‐N‐acetyltransferase (AANAT) and is subsequently methylated to melatonin by hydroxyindole‐O‐methyltransferase (HIOMT) (Hardeland and Poeggeler 2012). Melatonin is lipophilic and is, therefore, able to pass directly through cell membranes (Yu et al. 2016) and enter the nucleus (Acuña‐Castroviejo et al. 2014). It can also act through melatonin receptors, which are membrane‐bound and G‐coupled receptors(Hardeland and Poeggeler 2012; Sarlak et al. 2013; Takumida and Anniko 2019). Melatonin interacts with cytosolic proteins and binds to transcription factors (Hardeland and Poeggeler 2012; Acuña‐Castroviejo et al. 2014). In addition, it has direct free radical scavenging properties (Sarlak et al. 2013) and stimulates antioxidant enzyme production (Hardeland and Poeggeler 2012; Sarlak et al. 2013). This neurohormone is not only relevant for neurodegenerative processes but also for neurogenesis (Hardeland and Poeggeler 2012; Acuña‐Castroviejo et al. 2014). Similar to melatonin, the intermediate NAS has antioxidative and anti‐inflammatory effects and, furthermore, is an agonist for the tropomyosin receptor kinase B (TrkB), a neurotrophic growth factor receptor (Oxenkrug 2005). Therefore, in addition to melatonin, the synthesis pathway and the localization of its enzymes are also of significant importance.

The current study specifically targets a commonly overlooked life stage, early postnatal rats prior to the onset of auditory function. The aim of this study is to investigate developmental differences in the expression of melatonin and its synthesizing enzymes (AANAT and HIOMT) in the cochlea of postnatal and adult rats.

At postnatal stage, the cochlear architecture is still under construction. The inner sulcus is filled by Kölliker's organ, and the minor tectorial membrane is located above the hair cells (Roth and Bruns 1992). Claudius, Böttcher, and Hensen cells are undifferentiated and form a single epithelial layer (Roth and Bruns 1992). Four‐day‐old rats have only small quantities of fibrocytes in the spiral ligament (Zuo and Rarey 1997). Investigating melatonin's expression at this point not only fills a significant gap in the existing scientific literature but also has the potential to recalibrate current understanding of its multi‐faceted roles in cochlear health and development.

The current study examined the expression patterns of melatonin and its synthesizing enzymes in postnatal and adult stages in rat cochleae. It pinpoints regions of melatonin synthesis and accumulation, providing new insights into its role in cochlear protection and development.

## 2 Methods

### 2.1 Animals

Sprague Dawley rats (Charles River, Wilmington, USA) of three different age stages were used for the experiments: postnatal day 4 rats (*n* = 5, three males, two females), young adult rats at 6 weeks of age (*n* = 2, one male, one female), and 7‐month‐old adult rats (*n* = 5, two males, three females). The cochleae analyzed were from at least three animals of different parents, litters, and both sexes, with the exception of the supplemental group of 6‐week‐old animals. Postnatal day 4 rats were decapitated. Adults were euthanized with carbon dioxide. The animals were euthanized during the daytime. The used euthanization techniques and the number of euthanized animals were reported to the local authorities who approved our procedures. All experiments were conducted in accordance with the Animal Welfare Act as well as the German regulations, in compliance with the EU‐Directive 2010/63 on the protection of animals used for scientific purposes, specifically following the §4(3) of the German Animal Welfare Act.

### 2.2 Animal Preparation and Fixation

The animals were euthanized in the morning between 10 and 11 AM. The preparation was performed according to Sobkowicz et al. (1993). Skulls were kept on ice until they were transferred to a Petri dish containing Hank's balanced salt solution (HBSS; Gibco/Thermo Fisher, Kandel, Germany). Cochleae and vestibular organs were separated under the stereomicroscope (Zeiss, Oberkochen, Germany) and stored for further use. A small hole was made in the apex of the cochlea using forceps. Subsequently, specimens were fixed for 90 min in paraformaldehyde (Electron Microscopy Sciences, Hatfield, UK) diluted to 4% (v/v) in 0.1 M sodium phosphate buffer, pH 7.3 (Merck, Darmstadt, Germany) at room temperature, followed by washing in Tris buffered saline, pH 7.6 (TBS; Appli Chem, Darmstadt, Germany).

### 2.3 Decalcification and Cryosectioning

Decalcification was performed in 0.35 M ethylendiamintetraacetate (Sigma/Merck) containing Dulbecco's phosphate‐buffered saline, pH 7.3 (Gibco) (EDTA‐DPBS): postnatal day 4 specimens were incubated for 10 days, adult specimens for 2 weeks. Following decalcification and rinsing with DPBS, the specimens were incubated in ascending concentrations of 10%, 17%, 20%, and 23% (w/v) sucrose solution (Sigma) for 30 min at each step at room temperature. The specimens were transferred to 30% (w/v) sucrose solution and stored overnight at 4°C. Subsequently, the specimens were immersed in Tissue‐Tek (Sakura, Alphen aan den Rijn, Netherlands) and degassed in a desiccator. Thereafter, they were carefully aligned and frozen. Serial sections with a thickness of 7 µm were prepared with a cryostat (Thermo Fisher) at −25°C and mounted onto Superfrost Excell Adhesion microscope slides (Menzel/Epredia, Braunschweig, Germany).

### 2.4 Immunohistochemistry

The sections were dried at room temperature for 1 h. Subsequently, the Tissue‐Tek was removed by rinsing with washing buffer (DCS, Hamburg, Germany). Enzyme‐blocking was performed by incubating the sections with Dual Endogenous Enzyme Block (Dako, Carpinteria, USA) for 10 min. The blocking of unspecific antigen‐sites was performed by incubating in DPBT buffer containing 5% (v/v) goat serum (Sigma), 1% (w/v) bovine serum albumin (BSA; Roth, Karlsruhe, Germany), and 0.1% (v/v) Triton X (Sigma) for 30 min. Between these steps, the sections were rinsed in washing buffer. Primary antibodies were diluted in Antibody Diluent (Dako): HIOMT (SAB1303201, rabbit, 1/50; Sigma) followed by incubation of the slides for 1 h at room temperature. The slides were subsequently incubated overnight at 4°C either in AANAT antibody (CSB‐PA231814, rabbit, 1/50; Cusabio, Houston, USA), in melatonin antibody (MBS2006583, rabbit, 1/200, MyBioSource, San Diego, USA), or in IgG rabbit antibody (919.831.2240, 1/80, ImmunoReagents, Raleigh, USA), respectively. DCS detection line staining kits (Super Vision, HRP‐polymer rabbit, PD000EP‐K and PD00POL‐K, DCS) were used according to the manufacturer's instructions. For staining, the sections were incubated for 10 min in diamino‐benzidine (DAB) (DC137C100; DCS). Slides were mounted with Dako Mounting Medium (Dako). Photos were taken with a Zeiss Axioplan 2 (AxioCam ICc 1 camera; Zeiss) and processed with the Zeiss ZEN blue 2.1 lite software. Areas of the cochlea that were well‐prepared and adequately stained were analyzed by direct observation through the microscope ocular. The study focused on examining the actual expression in different age stages. Similar observations in the expression patterns of AANAT, HIOMT, and melatonin among individual animals within each age group were reported. For presentation purposes, microscopic representative images were processed as follows: color tone was white‐balanced with the background, and the exposure was increased. For microscopic images with higher magnification, particularly to visualize glia, the contrast was also enhanced.

## 3 Results

### 3.1 Age‐Dependent Expansion and Redistribution of Melatonin and Its Metabolizing Enzymes in the Cochlear Lateral Wall

**FIGURE 1:**
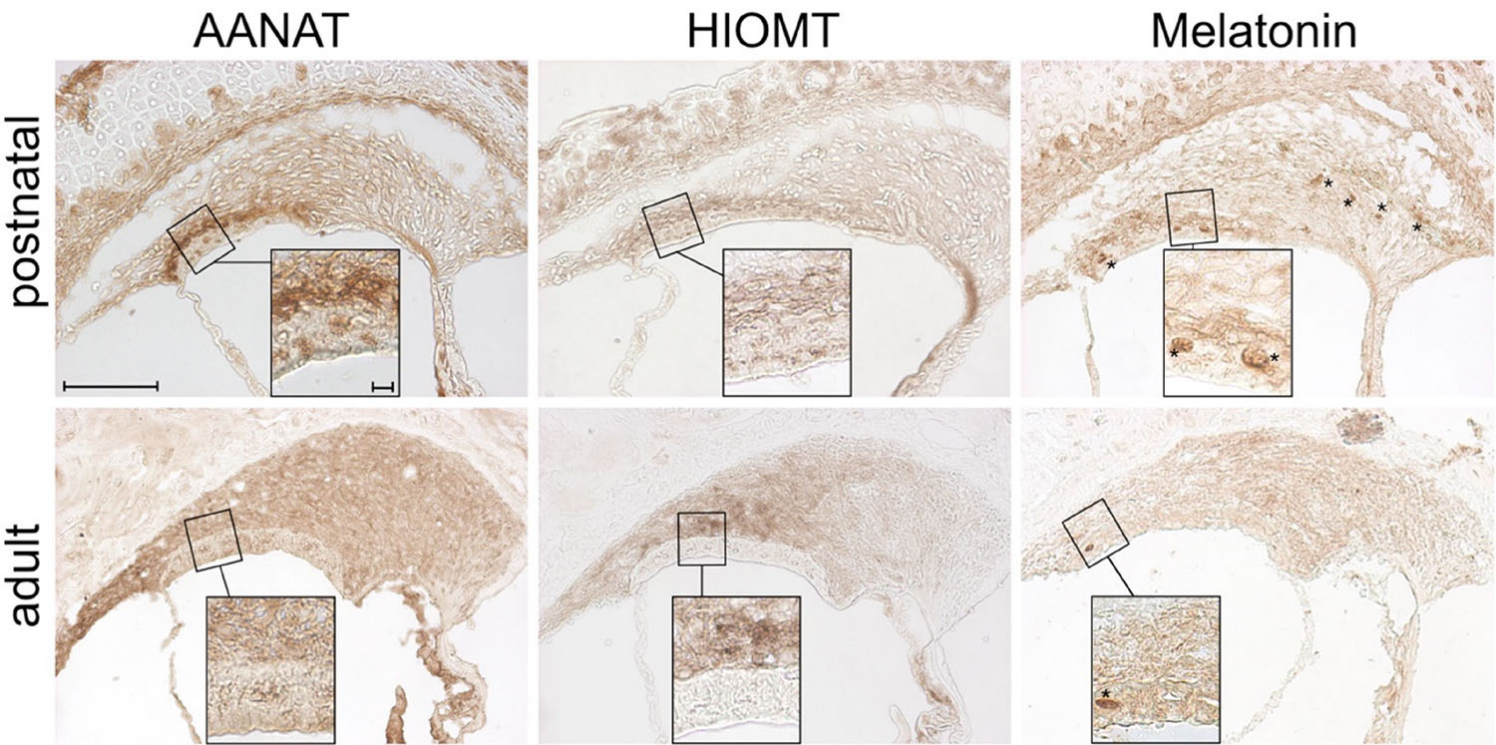
Expression of the enzymes AANAT and HIOMT and the accumulation of melatonin in the lateral wall in postnatal (4‐day‐old) and adult rats (7‐month‐old). In postnatal rats, AANAT and HIOMT were co‐expressed in the *stria vascularis*, the boundary layer between the *stria vascularis* and the spiral ligament and the otic capsule close to the spiral ligament. Melatonin accumulation was observed in the same regions and the blood vessels of the *stria vascularis*. The adult rats expressed AANAT in the *stria vascularis* and large parts of the spiral ligament, and strongly expressed HIOMT in the spiral ligament fibrocytes type I+V; melatonin accumulation in the entire spiral ligament and vessels or blood cells (indicated by asterisks) of the *stria vascularis* was also detected. Cochlear medial turn, scale bar: 100 µm. Inset: additional image of the type I spiral ligament fibrocytes adjacent to the *stria vascularis*; scale bar 10 µm (inset).

Both postnatal and adult rats displayed expression of AANAT and HIOMT enzymes in the lateral wall of the cochlea (Figure 1). The levels and areas of expression varied between these two developmental stages. No differences between sexes were apparent in the different age stages examined.

In postnatal rats, AANAT and HIOMT co‐expression was localized in the stria vascularis interspace, as well as in fibrocytes at the boundary between stria vascularis and spiral ligament. Outer sulcus cells in postnatal specimens did not express any of the markers examined. The postnatal extracellular spiral ligament layer near the otic capsule expressed AANAT and melatonin but lacked HIOMT. Moreover, the otic capsule cartilage adjacent to the spiral ligament was immunopositive for both enzymes and melatonin. Melatonin showed strong expression in postnatal blood vessels in stria vascularis and spiral ligament.

In adults, AANAT expression was observed in the stria vascularis' extracellular interspace and expanded to include the entire spiral ligament and outer sulcus cells. HIOMT exhibited a gradient of expression, particularly high around fibrocytes close to the adult stria vascularis (type I) and in the suprastrial region (type V). Other adult fibrocyte types and outer sulcus cells showed low‐to‐negative HIOMT expression. Melatonin was prevalent throughout the adult spiral ligament, outer sulcus cells, stria vascularis extracellular interspace, and blood vessels.

In comparison to postnatal rats, adult rats exhibited a broader spatial distribution of AANAT and HIOMT expression, most notably expanding into the entire spiral ligament and outer sulcus cells, illustrating an age‐dependent expansion and redistribution. Additional evidence was provided through staining in 6‐week‐old adult rats, shown in Figures S1–S2. Negative controls for the cochlear immunostaining are presented in Figure S3.

### 3.2 Age‐Associated Shift of Marker Expression From Extracellular to Intracellular Expression of Epithelial Supporter Cells in the Sensory Epithelium

**FIGURE 2:**
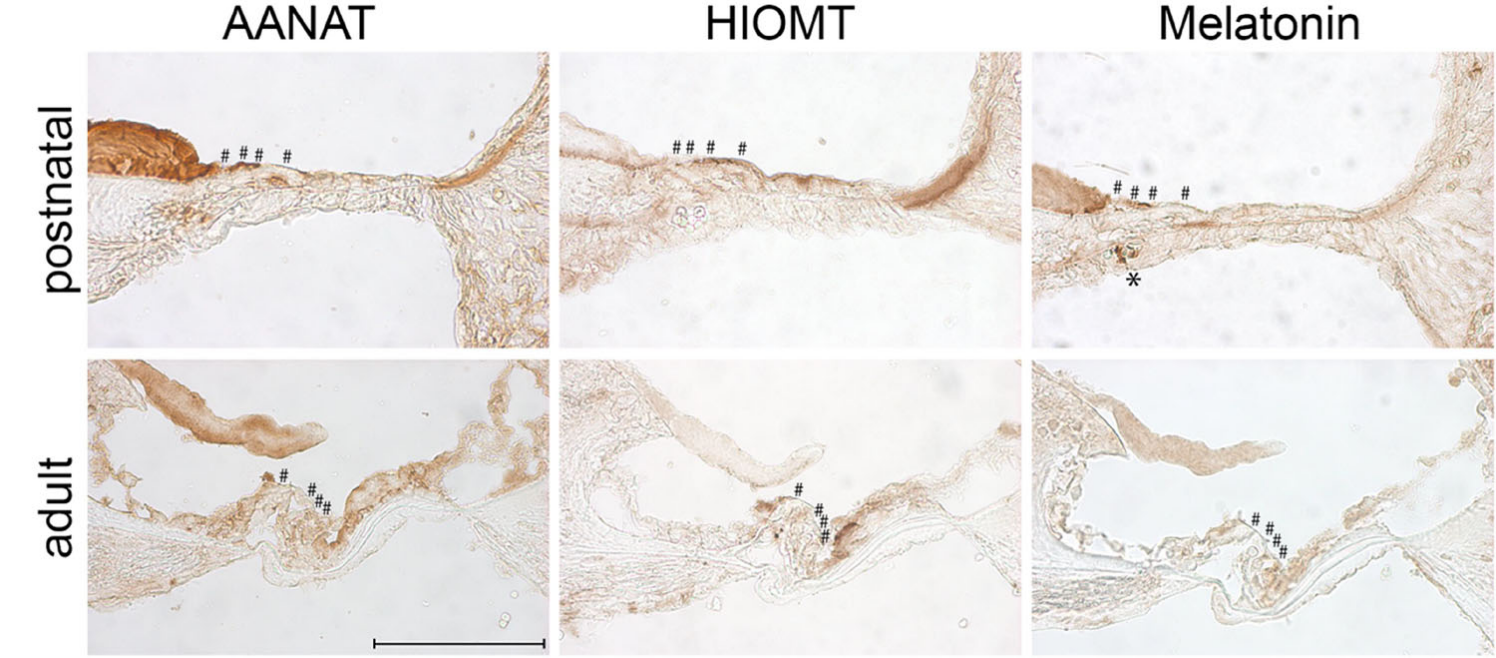
Expression of AANAT, HIOMT, and melatonin in the epithelial ridge of postnatal rats (4‐day‐old) and in the Corti organ of adults (7‐month‐old). In postnatal rats, all markers were expressed in the minor tectorial membrane and in the extracellular membrane above and below the epithelial supporter cells, as well as above and below Kölliker's organ. Most of the tectorial membrane was negative for HIOMT. AANAT, HIOMT, and melatonin expression was detected in the undifferentiated sensory epithelium near the hair cells. Melatonin accumulation was observed in a blood vessel (indicated by the asterisk). In adults, AANAT, HIOMT, and melatonin‐positive cells were observed in the epithelium (Hensen, Claudius, and inner border cells) surrounding the hair cells. AANAT was expressed in the adult inner sulcus cells. Hair cells were negative for all markers at both ages (below hashtags). Cochlear medial turn, scale bar 100 µm.

In the present study, as shown in Figure 2, we observed a significant age‐related shift in the expression of the markers AANAT, HIOMT, and melatonin, specifically within the epithelial supporter cells of the sensory epithelium.

In postnatal rats, the minor tectorial membrane, as well as the areas surrounding the hair cells and the Kölliker's organ, displayed positive expression for all three markers: AANAT, HIOMT, and melatonin. Within the Kölliker's organ itself, particular regions exhibited the expression of AANAT. Interestingly, both the postnatal Hensen and Claudius cells showed extracellular expression of AANAT and HIOMT above and below these cells. Furthermore, the basilar membrane in postnatal rats was found to be positive exclusively for melatonin.

Transitioning to adult rats, we noticed that the expression pattern of these markers shifted from extracellular expression in the postnatal rats to intracellular expression in the adults. Adult inner border cells demonstrated strong intracellular expression of all markers. The inner sulcus cells were unique in that they expressed AANAT but were devoid of HIOMT in adult rats. The adult basilar membrane was also negative for them. Neither the hair cells nor the phalangeal cells expressed any of the markers at both ages. Both the Hensen and Claudius cells revealed high intracellular levels of all markers.

Across all age groups, the tympanic covering layer consistently showed no expression for any of the markers in question.

In postnatal rats, marker expression was predominantly extracellular, whereas in adult rats, the expression shifted toward an intracellular pattern. Particularly, the border cells of the sensory epithelium indicated this age‐associated transition.

### 3.3 Spiral Limbus Fibrocytes Expressed AANAT and HIOMT Across Life Stages

**FIGURE 3:**
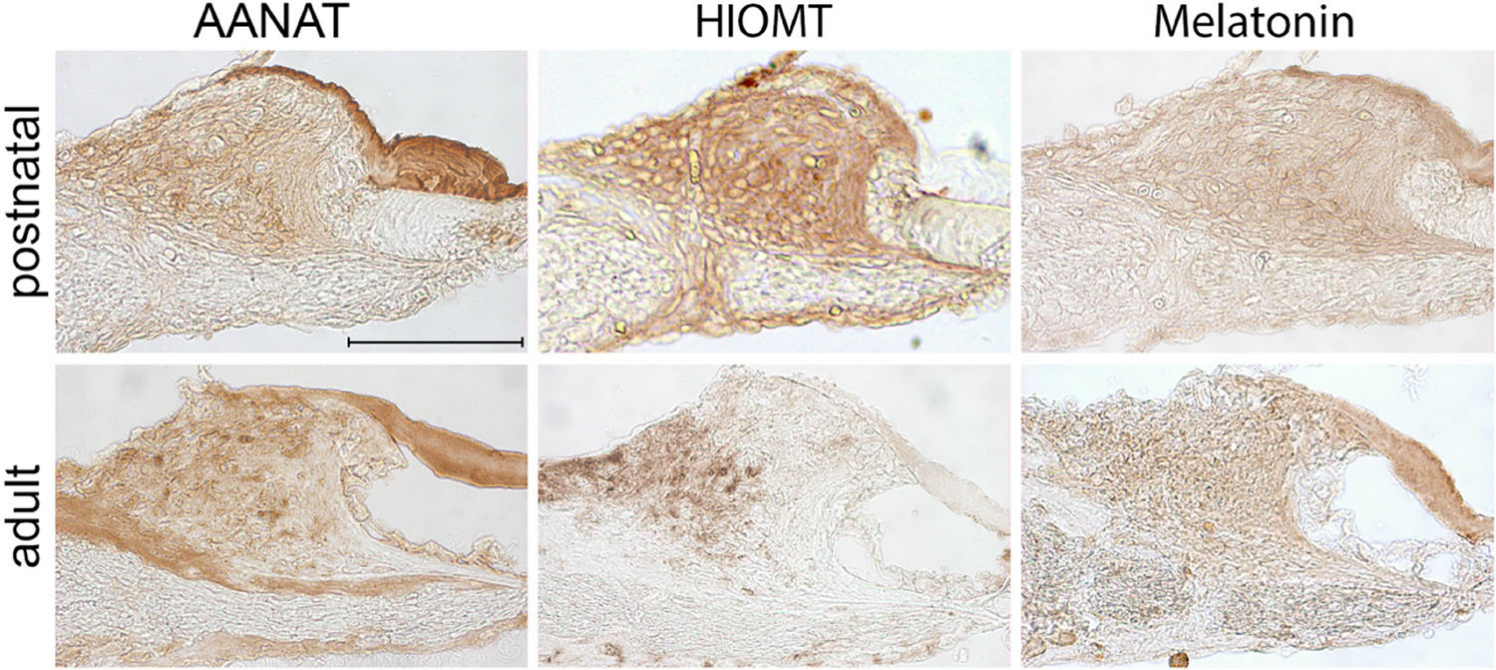
Expression of AANAT, HIOMT, and melatonin in the limbus. In and around the fibrocytes of the limbus of both ages, AANAT and HIOMT were detected in addition to melatonin. The tectorial membrane of the postnatal rats (4‐day‐old) was positive for AANAT and melatonin, while HIOMT was expressed above the interdental cells. The tectorial membrane of the adult rats (7‐month‐old) was also positive for AANAT and melatonin but entirely negative for HIOMT. Cochlear medial turn, scale bar 100 µm.

As depicted in Figure 3, both postnatal and adult rats demonstrated the expression of melatonin, AANAT and HIOMT within and surrounding the fibrocytes of the spiral limbus.

In postnatal rats, another region of focus was the extracellular matrix situated above the interdental cells and the Kölliker's organ. This area tested positive for all three markers . Conversely, the remaining portion of the tectorial membrane was only positive for AANAT and melatonin but lacked HIOMT expression.

**FIGURE 4:**
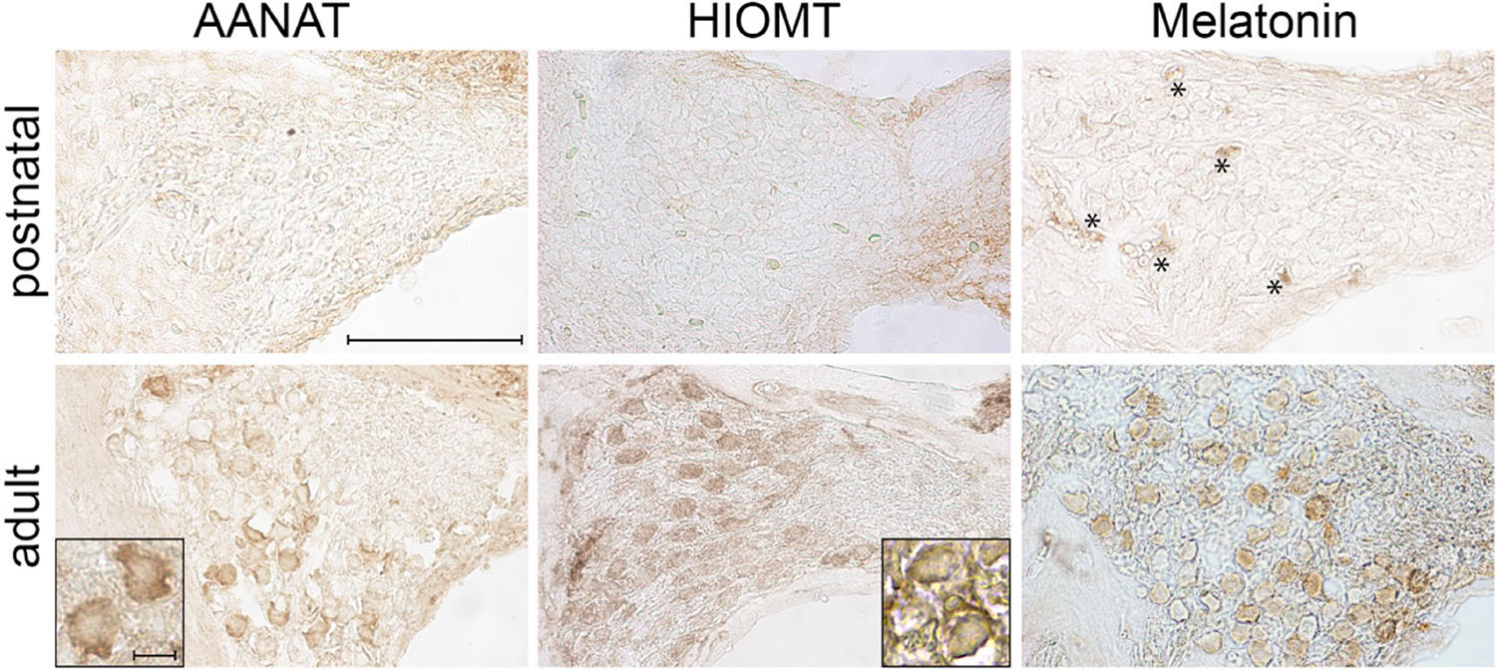
Expression of AANAT, HIOMT, and melatonin in the spiral ganglion. Postnatal rats (4‐day‐old) none of the markers were expressed. Adult rats (7‐month‐old): expression of AANAT, HIOMT, and an accumulation of melatonin in the neurons and satellite glia. Melatonin was accumulated in the vessels and blood cells of both ages (indicated by asterisks). Cochlear medial turn, scale bar 100 µm; inset:magnified image of spiral ganglion neurons with satellite glia, scale bar 5 µm.

In adult rats, a noteworthy observation was the age‐related decline in HIOMT expression, contrasting with the more stable levels of AANAT and melatonin. The interdental cells displayed no presence of either enzyme or melatonin in both ages. On the contrary, the tectorial membrane was consistently positive for AANAT and melatonin.

In both postnatal and adult rats, AANAT and HIOMT were expressed in the spiral limbus fibrocytes, but adult rats showed an age‐related decrease in HIOMT expression, contrasting with the more stable levels of AANAT.

### 3.4 Exclusive Expression of Melatonin, AANAT, and HIOMT in the Adult Rat Spiral Ganglion

Detailed in Figure 4, the spiral ganglion in postnatal rats exhibited a specialized melatonin presence, confined solely to the vasculature. In contrast, adult rats displayed more extensive expression profiles. Both melatonin and its key synthesizing enzymes, AANAT and HIOMT, were localized in the neurons and adjacent satellite glia within the spiral ganglion.

Notably, this adult neuronal expression was specific to the somatic portion of the neurons; neither the nerve fibers nor the Schwann cells around them were immunopositive for these markers.

**FIGURE 5:**
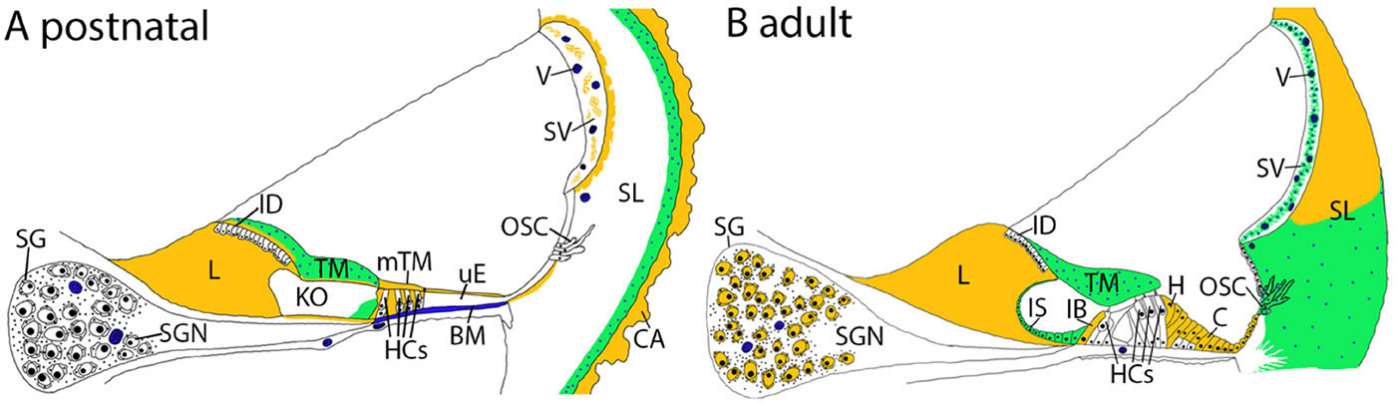
Overview of melatonin synthesis in the cochlea of postnatal (A) and adult (B) rats. Areas of presumed melatonin synthesis are shown in orange. Locations of presumed incomplete melatonin are colored in green (AANAT positive, HIOMT negative). Additional melatonin expression was detected in the areas stained blue or the blue spotted areas. In the spiral ligament and sensory epithelia, presumed melatonin synthesis was stronger and more widespread in the adults than in the postnatal rats. However, AANAT, HIOMT, and melatonin were particularly prominent in the extracellular matrix of postnatal rats. In the spiral ganglion, only the adults expressed AANAT, HIOMT, and melatonin. Abbreviations: BM, basilar membrane; C, Claudius cell; CA, otic capsule cartilage; IB, inner border cell; ID, interdental cell; H, Hensen cell; HCs, hair cells; IS, inner sulcus cell; L, spiral limbus; KO, Kölliker's organ; mTM, minor tectorial membrane; OSC, outer sulcus cell; SG, satellite glia; SV, stria vascular; SL, spiral ligament; SGN, spiral ganglion neuron; TM, tectorial membrane; uE, undifferentiated epithelial cells; V, vessel. Illustration of the medial turn. More details in Supplementary Table 1.

While melatonin in postnatal rats was limited to vascular areas, adult rats exhibited a more diversified pattern, with melatonin and its synthesizing enzymes, AANAT and HIOMT, present in the neurons and adjacent satellite glia, indicating an age‐related specialization. A graphical representation and summary of the observed expression of postnatal and adult rats are shown in Figure 5.

## 4 Discussion

In the revised investigation, the research illuminates the relatively understudied role of melatonin in the cochlear auditory functions across different life stages, specifically in adult and postnatal rats. The study's significant insights bridge this gap in knowledge, offering implications for not just rats, but potentially extending to other species, including humans. Reduced melatonin synthesis in postnatal rats, especially in the spiral ligament and ganglion, presents a contrast to the levels found in adults. Rats that are 4 days old exhibit characteristics such as deafness, an undifferentiated Corti organ, and a lack of endocochlear potential (Chen and Zhao 2014; Lautermann et al. 1999).

At 4 days, rat development seemed to mirror human embryos at 10–12 weeks (Roth and Bruns 1992; Locher et al. 2013; Payne 2006; Graven 2008) with comparable melatonin reductions. Between 6 weeks and 7 months, adult rats showed consistent melatonin synthesis reminiscent of post‐pubertal peaks in humans (Payne 2006; Noriega et al. 2009), suggesting that melatonin synthesis does not decline in young rats, reflecting the pattern observed in humans.

Our study enhances the understanding of melatonin synthesis in adult animals, building on prior findings of its presence in various cochlear regions of guinea pigs (Biesalski et al. 1988), rats (López González et al. 1998), and mice (Takumida and Anniko 2019). Unlike earlier research, we discovered that hair and pillar cells lack melatonin, with its synthesizing enzymes found only in the soma of adult spiral ganglion neurons.

This precise localization of melatonin synthesis, provided by identifying AANAT and HIOMT enzymes, offers detailed insights into melatonin accumulation in the cochlea.

In postnatal rats, melatonin, AANAT, and HIOMT expression was elevated in extracellular areas such as the stria vascularis and spiral ligament interfaces. Melatonin exerts certain functions independently of membrane receptors. Melatonin, itself, has a direct free radical scavenging activity (Reiter et al. 2000). The present study demonstrates the possibility that extracellular melatonin synthesis occurs in the spiral limbus, spiral ligament, postnatal tectorial membrane (above the interdental cells and Kölliker organ), the minor tectorial membrane, extracellular matrix of supporter cells of the undifferentiated epithelia, and in postnatal stria vascularis extracellular interspace.

Further in vitro studies have revealed that melatonin accumulates in the extracellular matrix or scaffolds that mimic the extracellular matrix and that it has a beneficial effect on neighboring cells (He et al. 2014; Kaczmarek‐Szczepańska et al. 2021). He et al. (2014) tested the binding of melatonin to the extracellular matrix of bone marrow‐derived mesenchymal stem cells and showed an increase in their proliferation and a reduction of intracellular oxidative stress. Kaczmarek‐Szczepańska et al.’s (2021) study used a collagen–chitosan matrix as the melatonin carrier that elevated the cell viability of human epidermal keratinocytes, dermal fibrocytes, and melanoma cells.

Apart from the aforementioned findings, melatonin in the extracellular matrix may also directly affect the enzymatic function of metalloproteases (MMPs) which play a role in the remodeling of the extracellular matrix (Rudra et al. 2013, Ganguly and Swarnakar 2009). Rudra et al. (2013) showed in a gelatin zymographic analysis a reduction of active MMP‐9 in the presence of melatonin. In a computer simulation, the protein sequences of MMP‐9 and melatonin revealed potential interaction sites (Rudra et al. 2013). Ganguly showed a direct enzymatic downregulation of secreted MMP‐3 and MMP‐9 by melatonin using gelatin and casein zymography in mouse gastric ulcer healing (Ganguly and Swarnakar 2009). In this study, melatonin, as well as its synthesizing enzymes, were detected in extracellular locations. The idea that melatonin or its intermediates might be synthesized extracellularly represents a fundamentally new concept that requires further investigation in future studies.

An intriguing discovery is the notable expression of melatonin and its synthesizing enzymes in the ear capsule, which remains incompletely ossified at the postnatal stage (Richard et al. 2017). This points to a potential role for melatonin in cartilage and bone development (Zhang et al. 2022) in the ear capsule.

The current study observed that the inner ear of postnatal rats also expressed melatonin within the vessels. Although the pineal gland does not yet produce melatonin at this stage, melatonin is produced extrapineally in a variety of cells, in particular the platelets, erythrocytes, and endothelia (Acuña‐Castroviejo et al. 2014). The extrapineal production is not subject to the circadian cycle. Although pineal melatonin does indeed reach the cochlea at night (Jang et al. 2010), the organs were collected in the late morning. Therefore, the detected melatonin was either stored or produced extrapineally. Our results demonstrate the expression of melatonin‐synthesizing enzymes during the day, suggesting that melatonin may also be produced during the daytime.

Additionally, there are other external sources, such as nocturnal breast milk, comparable to those in humans (Acuña‐Castroviejo et al. 2014). Such a supply, particularly via the bloodstream, could offer essential protection against ototoxic damage in postnatal rats. Administering melatonin externally and transport via the bloodstream could also be a viable route of delivery for human fetuses, effectively targeting the cochlea.

The study identifies an age‐related expansion of presumed NAS expression in the cochlear lateral wall, where AANAT was present, but HIOMT was absent. NAS is a precursor of melatonin and is synthesized from serotonin by AANAT. In addition to its role as an antioxidant and anti‐apoptotic substance, NAS may also act as a TrkB agonist (Jang et al. 2010). In a BDNF knockout model of the mouse hippocampal neurons, in both in‐vivo and in vitro studies, NAS was able to activate TrkB and downstream signaling. Yu et al. (2016) have shown in a membrane permeability measurement on a human kidney cell line that NAS has an even higher permeability than melatonin. Despite these studies, the cochlear function of NAS is completely unknown; thus, the present study provides an approach for further research.

The current study provides key insights into melatonin synthesis and distribution in the cochlea, highlighting age‐related increases in assumed synthesis sites. Despite relying on immunohistochemical analysis, the presence of enzymes in certain cells and extracellular areas indicates endogenous cochlear melatonin synthesis. It reveals a shift from extracellular to intracellular melatonin localization, underscoring the need for further research. By pinpointing cells and areas involved in melatonin production, the study paves the way for treatment strategies and insights into hearing development, while also encouraging comparative research across species.

## Author Contributions

Monika Orsolic: Data curation, formal analysis, investigation, methodology, validation, visualization, writing–original draft. Marc Diensthuber: Data curation, writing–review and editing. Timo Stöver: Resources, writing–review and editing, supervision. Christin Geißler: Conceptualization, data curation, formal analysis, project administration, validation, visualization, writing–original draft.

## Conflicts of Interest

The authors declare no conflicts of interest.

## Supporting information




**Supplemental Table 1: Summary of AANAT, HIOMT and melatonin expression in postnatal and adult rats**. Presumed melatonin synthesis due to co‐localization of AANAT and HIOMT (orange letters) in the same area or cells. Presumed NAS synthesis due to localization of AANAT but not HIOMT (green letters). Apart from the areas of presumed melatonin synthesis indicated by the co‐localization of AANAT and HIOMT, singular melatonin expression (blue letters) was found in some areas. Extracellular expression is marked with asterisks (*). Abbreviations: *Stria vascularis* (SV), spiral ligament (SL), melatonin (Mel).
**Supplemental Figure 1: Overview of the staining for AANAT, HIOMT and melatonin in the cochlea of postnatal (4‐day‐old) and adult rats (6‐week‐old and 7‐month‐old)**. Under low magnification, the age‐independent expression of AANAT and melatonin could be seen in large parts of the tectorial membrane and the limbus. Although wide parts of the adult spiral ligament were positive for melatonin and its metabolizing enzymes. In postnatal rats, the triple‐positive staining was strong in the spiral ligament at the barrier to the *stria vascularis* and in the otic capsule close to the spiral ligament. In the spiral ganglion of adults, all markers were expressed, while this staining was absent in postnatal rats except for blood vessels. Cochlear medial turn, scale bar 100 µm.
**Supplemental Figure 2: Staining for AANAT, HIOMT and melatonin in the 6‐week‐old adult rats**. The staining pattern was comparable to that of the 7‐month‐old rats. Cochlear medial turn. Limbus, spiral ganglion, Corti organ, lateral wall, scale bar: 100 µm; enlarged section of the lateral wall, scale bar: 10 µm (inset).

**Supplemental Figure 3: Negative controls of the cochlear staining**. Immunohistochemical staining with IgG rabbit. No unspecific staining was detected. Cochlear medial turn, scale bar 100 µm.

## Data Availability

The datasets generated and analyzed during the current study are available from the authors upon reasonable request.
