# Supplementary material for: Upregulated Expression and Shifted Distribution of Melatonin and Its Synthesizing Enzymes From Postnatal to Young Adult Rat Cochleae
Source: Dev Neurobiol. 2025 Jun 18;85(3):e22979. doi: 10.1002/dneu.22979 (PMC12177440; doi:10.1002/dneu.22979)
Supplement: Supplementary file 1 — Supplemental Table 1: Summary of AANAT, HIOMT and melatonin expression in postnatal and adult rats. Presumed melatonin synthesis due to co‐localization of AANAT and HIOMT (orange letters) in the same area or cells. Presumed NAS synthesis due to localization of AANAT but not HIOMT (green letters). Apart from the areas of presumed melatonin synthesis indicated by the co‐localization of AANAT and HIOMT, singular melatonin expression (blue letters) was found in some areas. Extracellular expression is marked with asterisks (*). Abbreviations: Stria vascularis (SV), spiral ligament (SL), melatonin (Mel). Supplemental Figure 1: Overview of the staining for AANAT, HIOMT and melatonin in the cochlea of postnatal (4‐day‐old) and adult rats (6‐week‐old and 7‐month‐old). Under low magnification, the age‐independent expression of AANAT and melatonin could be seen in large parts of the tectorial membrane and the limbus. Although wide parts of the adult spiral ligament were positive for melatonin and its metabolizing enzymes. In postnatal rats, the triple‐positive staining was strong in the spiral ligament at the barrier to the stria vascularis and in the otic capsule close to the spiral ligament. In the spiral ganglion of adults, all markers were expressed, while this staining was absent in postnatal rats except for blood vessels. Cochlear medial turn, scale bar 100 µm. Supplemental Figure 2: Staining for AANAT, HIOMT and melatonin in the 6‐week‐old adult rats. The staining pattern was comparable to that of the 7‐month‐old rats. Cochlear medial turn. Limbus, spiral ganglion, Corti organ, lateral wall, scale bar: 100 µm; enlarged section of the lateral wall, scale bar: 10 µm (inset). [file DNEU-85-0-s002.docx]

| **cochlear localization** | | **postnatal** | **adult** |
| --- | --- | --- | --- |
| lateral wall | SV interspace* | Mel synthesis* | NAS synthesis*  Mel expression* |
|  | SL fibrocytes | Mel synthesis SV/ SL boarder*  NAS synthesis SL/ otic capsule boarder*  Mel expression SL/ otic capsule boarder* | Mel synthesis type I+V  NAS synthesis type II to IV  Mel expression |
|  | outer sulcus cells | none | NAS synthesis  Mel expression |
|  | otic capsule cartilage | Mel synthesis | none |
| sensory epithelia | hair cells | none | |
|  | basilar membrane | Mel expression* | none |
|  | Hensen cells | Mel synthesis in minor tectorial membrane*, extracellular matrix* of undifferentiated epithelia and hair cells  spot of NAS synthesis in Kölliker’s organ | Mel synthesis in epithelia supporter cells |
|  | Claudius cells |  |  |
|  | inner border cells |  |  |
|  | inner sulcus cells |  | NAS synthesis in inner sulcus cells |
| spiral limbus | limbus fibrocytes | Mel synthesis | |
|  | interdental cells | none | |
|  | tectorial membrane (major)* | Mel synthesis above interdental cells and Kölliker’s organ*  NAS synthesis*  Mel expression* | NAS synthesis*  Mel expression* |
| spiral ganglion | spiral ganglion neurons | none | Mel synthesis |
|  | satellite glia |  |  |
| vessels | | Mel expression | |

**Supplemental Table 1: Summary of AANAT, HIOMT and melatonin expression in postnatal and adult rats.** Assumed melatonin synthesis due to co-localization of AANAT and HIOMT (orange letters) in the same area or cells. Assumed NAS synthesis due to localization of AANAT but not HIOMT (green letters). Apart from the areas of assumed melatonin synthesis indicated by the co-localization of AANAT and HIOMT, singular melatonin expression (blue letters) was found in some areas. Extracellular expression is marked with asterisks (*). Abbreviations: *Stria vascularis* (SV), spiral ligament (SL), melatonin (Mel).


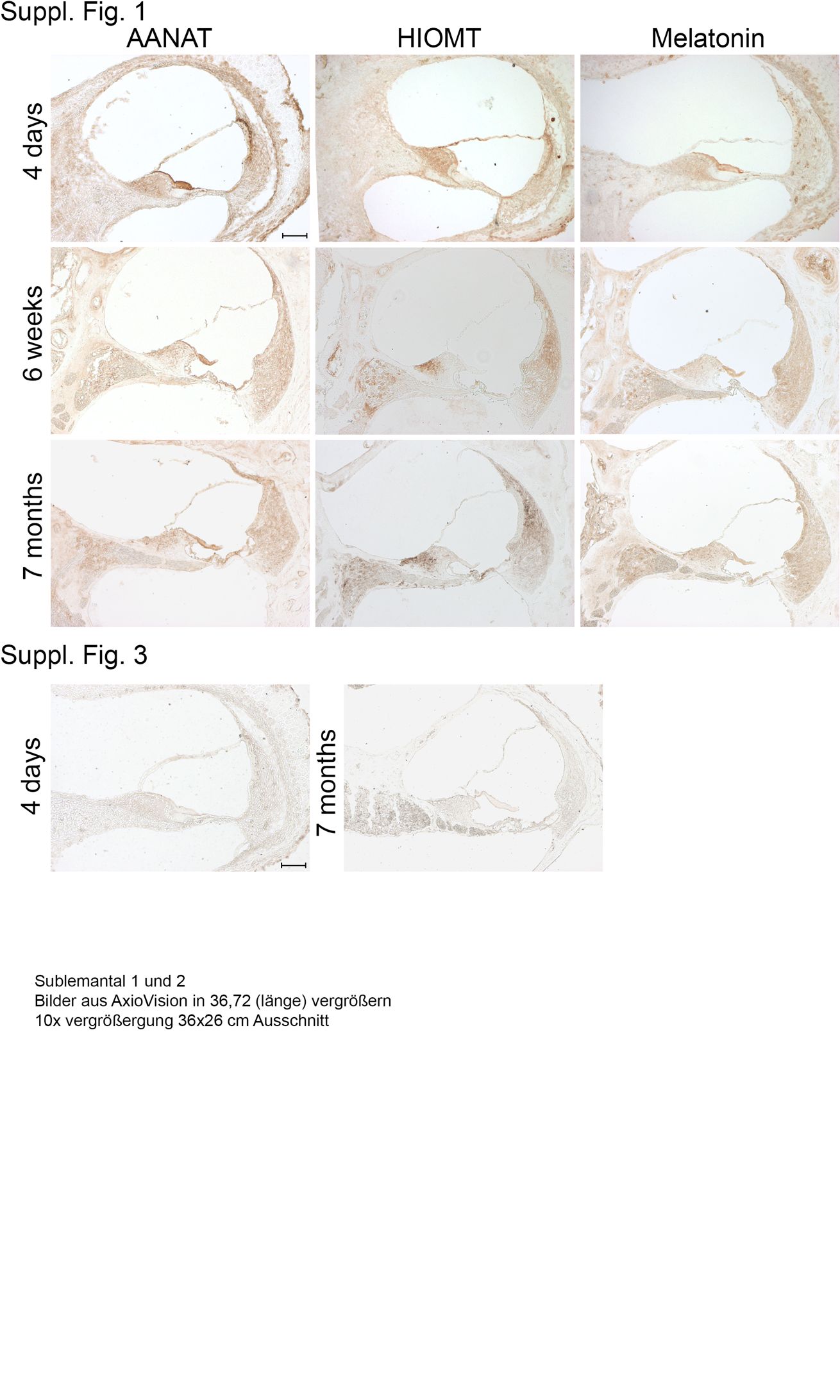


**Supplemental Figure 1: Overview of the staining for AANAT, HIOMT and melatonin in the cochlea of postnatal (4-day-old) and adult rats (6-week-old and 7-month-old).** Under low magnification, the age-independent expression of AANAT and melatonin could be seen in large parts of the tectorial membrane and the limbus. Although, wide parts of the adult spiral ligament were positive for melatonin and its metabolizing enzymes. In postnatal rats, the triple positive staining was strong in the spiral ligament at the barrier to the *stria vascularis* and in the otic capsule close to the spiral ligament. In the spiral ganglion of adults, all markers were expressed, while this staining was absent in postnatal rats except for blood vessels. Cochlear medial turn, scale bar 100 µm.


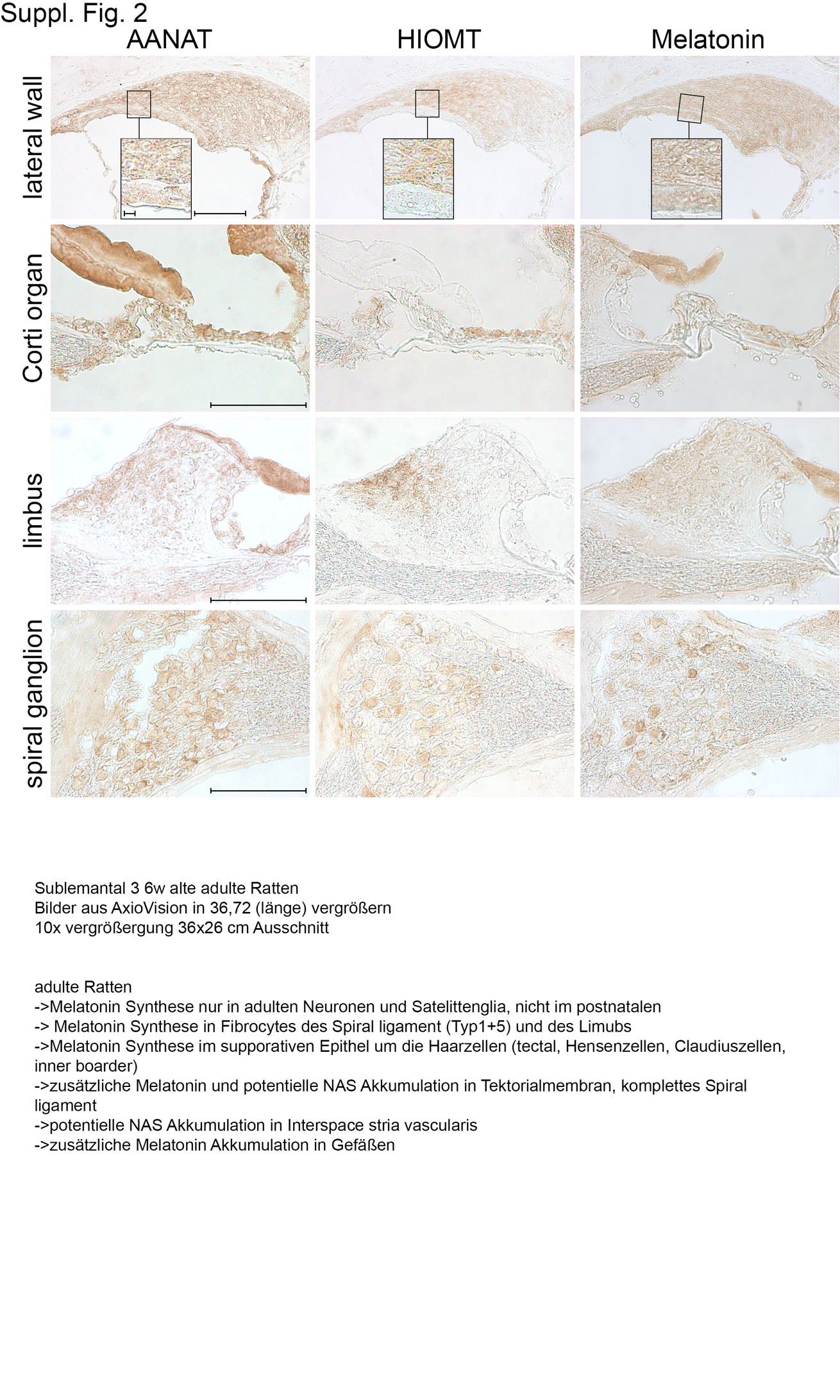


**Supplemental Figure 2: Staining for AANAT, HIOMT and melatonin in the 6-week-old adult rats.** The staining pattern was comparable to the 7-month-old rats. Cochlear medial turn. Limbus, spiral ganglion and the Corti organ scale bar 100 µm; lateral wall, scale bar 100 µm; enlarged section of the lateral wall, scale bar 10 µm (inset).


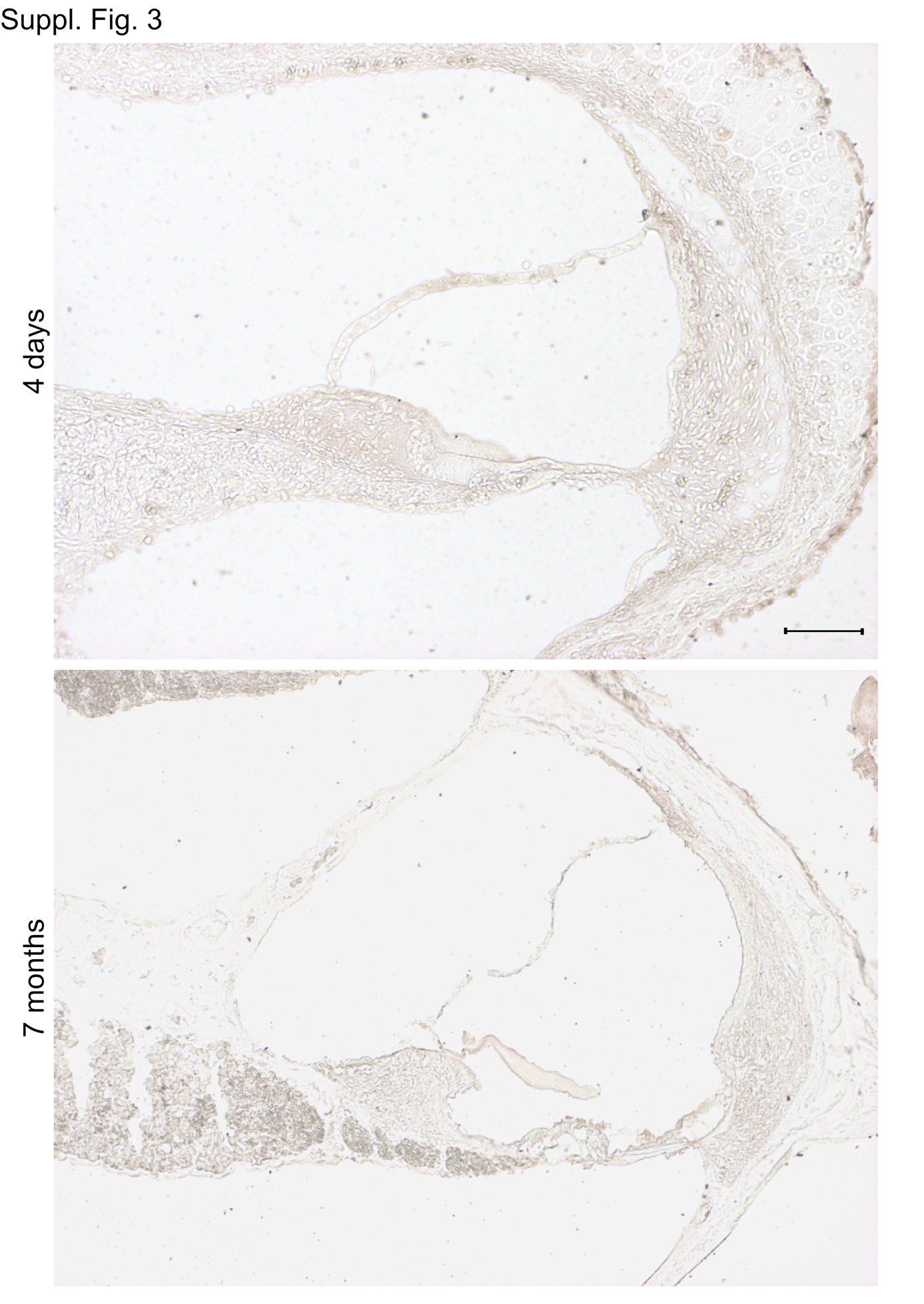


**Supplemental Figure 3: Negative controls of the cochlear staining**. Immunohistochemical staining with IgG rabbit. No unspecific staining was detected. Cochlear medial turn, scale bar 100 µm.
