# Supplementary figures and images for: Upregulated Expression and Shifted Distribution of Melatonin and Its Synthesizing Enzymes From Postnatal to Young Adult Rat Cochleae
Source: Dev Neurobiol. 2025 Jun 18;85(3):e22979. doi: 10.1002/dneu.22979 (PMC12177440; doi:10.1002/dneu.22979)

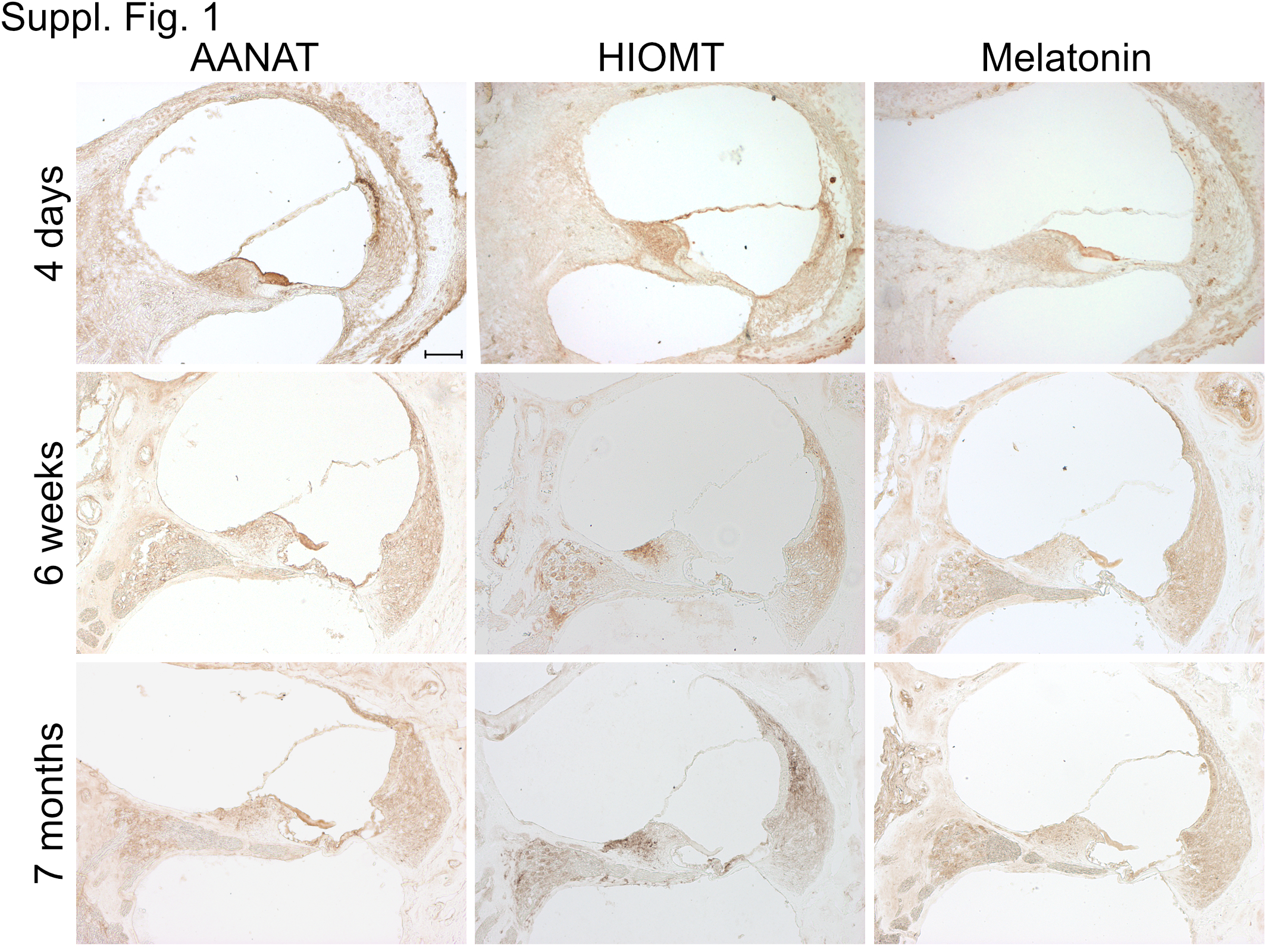

Supplement: Supplementary file 2 — Supplemental Figure 3: Negative controls of the cochlear staining. Immunohistochemical staining with IgG rabbit. No unspecific staining was detected. Cochlear medial turn, scale bar 100 µm. [file DNEU-85-0-s001.jpg]
